# Model-Based Design of Long-Distance Tracer Transport Experiments in Plants

**DOI:** 10.3389/fpls.2018.00773

**Published:** 2018-06-07

**Authors:** Jonas Bühler, Eric von Lieres, Gregor J. Huber

**Affiliations:** ^1^Plant Sciences (IBG-2), Institute of Bio- and Geosciences (IBG), Forschungszentrum Jülich, Jülich, Germany; ^2^Biotechnology (IBG-1), Institute of Bio- and Geosciences (IBG), Forschungszentrum Jülich, >Jülich, Germany

**Keywords:** tracer transport, phloem, modeling, PET, data analysis, ^11^C, design of experiments, experimental scheduling

## Abstract

Studies of long-distance transport of tracer isotopes in plants offer a high potential for functional phenotyping, but so far measurement time is a bottleneck because continuous time series of at least 1 h are required to obtain reliable estimates of transport properties. Hence, usual throughput values are between 0.5 and 1 samples h^−1^. Here, we propose to increase sample throughput by introducing temporal gaps in the data acquisition of each plant sample and measuring multiple plants one after each other in a rotating scheme. In contrast to common time series analysis methods, mechanistic tracer transport models allow the analysis of interrupted time series. The uncertainties of the model parameter estimates are used as a measure of how much information was lost compared to complete time series. A case study was set up to systematically investigate different experimental schedules for different throughput scenarios ranging from 1 to 12 samples h^−1^. Selected designs with only a small amount of data points were found to be sufficient for an adequate parameter estimation, implying that the presented approach enables a substantial increase of sample throughput. The presented general framework for automated generation and evaluation of experimental schedules allows the determination of a maximal sample throughput and the respective optimal measurement schedule depending on the required statistical reliability of data acquired by future experiments.

## Introduction

Studying long-distance transport in plants is of high interest for the investigation of functional traits under the influence of diverse environmental factors (Van Bel, 2003; Jahnke et al., 2009). Non-invasive methods using short-lived radioisotopes have been established to detect the transport of radioactive tracer *in vivo* (Jahnke et al., 1998, 2009; Minchin and Thorpe, 2003; Alexoff et al., 2011; Garbout et al., 2012; Weisenberger et al., 2013; Hubeau and Steppe, 2015; Nakanishi, 2017). For example, after labeling a plant with ^11^CO_2_, transported ^11^C can be detected and localized within the supplied plant organs from outside with positron emission tomography (PET) or scintillation detectors. These methods yield spatially and temporally resolved data of the tracer distribution within the plant, which can be analyzed by mathematical methods in order to estimate transport properties such as tracer transport velocities and leakage along the transport pathway (Tyree, 1975; Minchin and Thorpe, 2003; Suwa et al., 2008; Bühler et al., 2011, 2014, 2017; Converse et al., 2015). Further development of PET scanners dedicated for plant research (Streun et al., 2014, 2016) offers a high potential for plant phenotyping (Fiorani and Schurr, 2013; Hubeau and Steppe, 2015), but for this purpose the sample throughput needs to be increased. In view of the limited availability and high operating costs of radioisotope production and PET devices dedicated to plant research, a suitable way to increase throughput is by reducing the time of data acquisition per plant sample. Up to date, common tracer experiments in plants typically take at least 1 h of data acquisition plus sample handling times, limiting the throughput to not more than 1 sample per hour (Troughton et al., 1977; Jahnke et al., 2009; De Schepper et al., 2013; Converse et al., 2015). Increasing the throughput by simply shortening the data acquisition time will not work because information about transport properties as well as storage is typically spread over a large part of the time series (Bühler et al., 2011). Instead, the idea of the present study is to reach a higher sample throughput by introducing temporal gaps in the data acquisition of each sample, thus enabling to label multiple plants one after another with a temporal offset and to subsequently measure these plants in a rotating scheme. The resulting interrupted time series will always contain less information about the transport properties than a complete measurement. Thus the task was to identify experimental schedules regarding the distribution of gaps in data acquisition which maximize the throughput and at the same time minimize the loss of information. For this kind of evaluation, a model-based data analysis (Bühler et al., 2011, 2014) needs to be applied, because purely data driven methods (Minchin and Thorpe, 2003; Suwa et al., 2008; Converse et al., 2015) require uninterrupted time series. For the compartmental models established by Bühler et al. (2014), increasing gaps in the time series lead to higher uncertainties in the fitted model parameters. Thus the uncertainties can be used to rank experimental schedules according to the amount of lost information. To do this in a systematic way, a case study was set up, using a compartmental model and added noise to create data sets mimicking typical experimental data. These data sets were then used to evaluate a huge number of different experimental schedules. The working hypothesis was that the proposed scheme of interrupted time series created by rotational measurement should allow a substantial increase in sample throughput while maintaining a sufficient quality of data analysis.

## Numerical methods

### Model definition

The general class of compartmental tracer transport models was introduced in Bühler et al. (2014). This model class consists of up to *N* parallel compartments where all compartments are interconnected and allow exchange of tracer with individual exchange rates *e*_*ij*_ from compartment *i* to compartment *j*. Within the direction of spatial transport, tracer moves due to respective convection *v*_k_ and diffusion *d*_*k*_ in all compartments *k*, (*k* = 1… *N*). Accordingly, the underlying system of partial differential equations (PDEs) is defined as

(1) $$\frac{\partial \rho_{k}}{\partial t}=-v_{k}\frac{\partial \rho_{k}}{\partial x}+d_{k}\frac{\partial^{2}\rho_{k}}{\partial x^{2}}+\sum_{j=1}^{N}a_{kj}\rho_{j}$$

with ρ_*k*_ = ρ_*k*_(*x, t*) as tracer density distribution in space and time. The exchange between compartments *i* and *j* with exchange rate *e*_*ij*_ as well as the decay of tracer with decay rate λ is combined in *a*_*ij*_ as

$${\left(a_{ij}\right)}_{N\times N}=\left(\begin{array}{cccc} 0 & e_{12} & \cdots & e_{1N}\\ e_{21} & \ddots & & \vdots \\ \vdots & & \ddots & e_{(N-1)N}\\ e_{N1} & \cdots & e_{N(N-1)} & 0 \end{array}\right)-\left(\begin{array}{ccc} \sum_{n=1}^{N}e_{1n} & & 0\\ & \ddots & \\ 0 & & \sum_{n=1}^{N}e_{Nn} \end{array}\right)-\lambda I.$$

**Figure 1:**
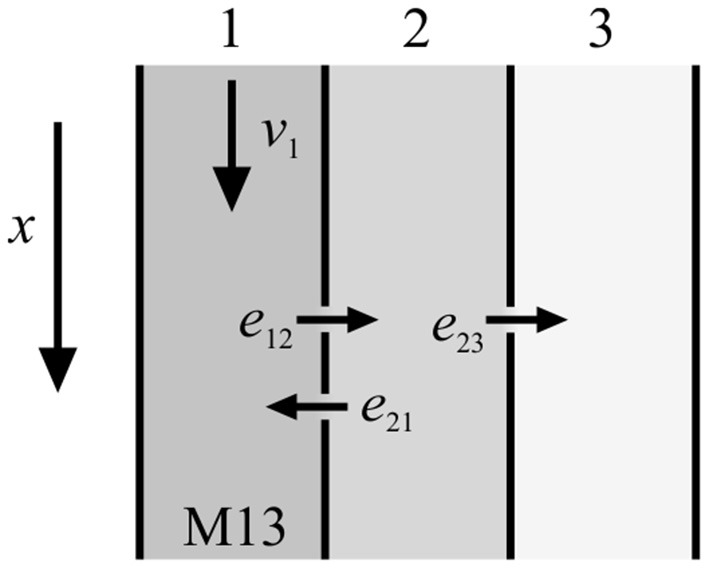
Sketch of model M13 from Bühler et al. (2014). There is transport in the first compartment with velocity *v*_1_, exchange and back-exchange from the first to the second compartment with exchange rates *e*_12_ and *e*_21_, respectively, and storage in the third compartment with exchange rate *e*_23_.

Tracer entering the system is described either by a spatial initial condition or by a time dependent boundary condition (Bühler et al., 2014). From this general model class single model instances can be derived by allowing only a certain set of model parameters to be non-zero. In Bühler et al. (2014), a model filter was presented which sorts out all redundant models as well as useless models that would have non-functional compartments. For up to 5 model parameters (without diffusion) a family of 48 individual models was established, sorted by complexity and numbered consecutively from M01 to M48. In the present study, mainly model M13 (Figure 1) was used for numerical calculations since it has been shown to be universally applicable to typical experimental data (De Schepper et al., 2013; Bühler et al., 2014).

This model consists of three compartments with four model parameters that characterize the transport properties. Convection is controlled by *v*_1_ and takes place in the first compartment only. Additionally, tracer can exchange (*e*_12_) and back-exchange (*e*_21_) from the first to the second compartment. Moreover, tracer can be stored in the third compartment (*e*_23_).

### Forward simulation and inverse problem

The PDE in Equation (1) was spatially discretized using finite volume schemes as described in Bühler et al. (2017). Here, a linear fifth order upwind scheme was used (Shu, 2009). This discretization method produces quite accurate results when the initial condition is sufficiently smooth, and, at the same time, is very quick to solve (Bühler et al., 2017). The spatial discretization results in a system of ordinary differential equations (ODEs) which then was solved with a standard fifth order Runge-Kutta time solver (Dormand and Prince, 1980). Finally, the result of a forward simulation is the sum of tracer over all compartments,

$$\rho \left(x,t\right)=\sum_{k=1}^{N}\rho_{k}\left(x,t\right).$$

The procedure of fitting the model to a given data set was described in Bühler et al. (2014). A nonlinear least squares optimization method was used for estimation of model parameters (lsqnonlin, Matlab R2016a, The MathWorks, Inc.).

### Parameter uncertainties

The model fitting yields estimates of the model parameters and the Jacobian matrix of the parameters at the optimum. Asymptotic standard errors of the parameter estimates were estimated from the square roots of the diagonal elements of the covariance matrix **Cov** of the model parameters. **Cov** was approximated by **Cov** = *s*^2^(**J**^T^**J**)−1, using the variance *s*^2^ of the fit and the Jacobian **J** of the estimated model parameters at the minimum (Johnson and Faunt, 1992). Though this method to estimate standard errors neglects the covariances between the estimated parameters, it is preferable here to the Monte Carlo bootstrap method used in Bühler et al. (2014), because it is computationally much less expensive and also applicable to the very small data sets considered in this study. Example comparisons showed that the results of both methods are very similar for large data sets used in this study.

### Implementation

All numerical routines have been implemented in MATLAB (R2016a, MathWorks, Inc.). The code is publicly available at https://github.com/ForschungszentrumJuelich/mdate.

## Experimental design

**Figure 2:**
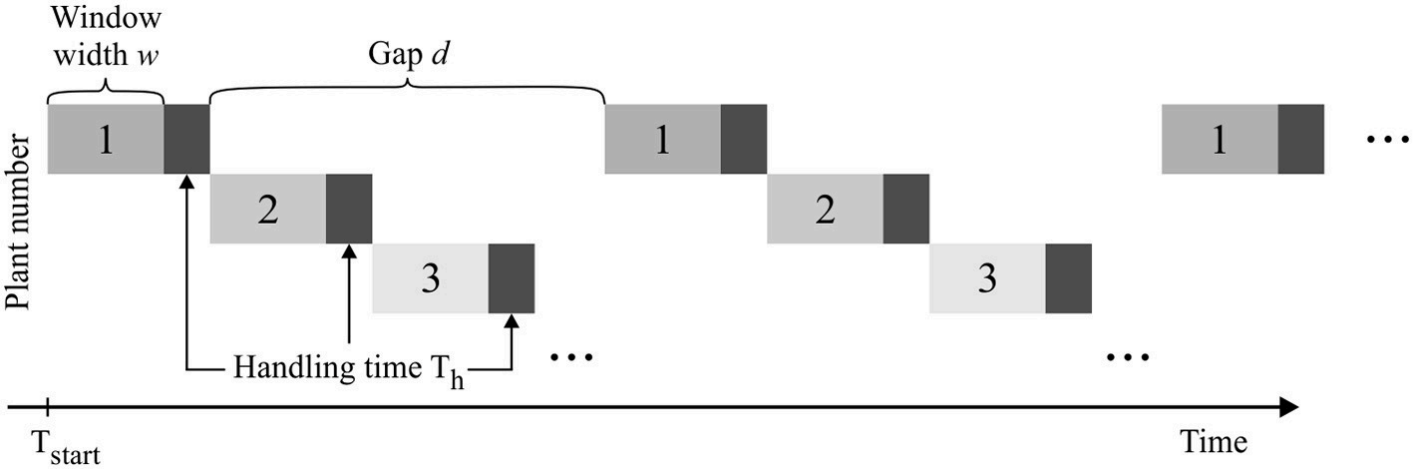
Consecutive measurement of multiple plant samples. For the pulse labeling experiment of plant 1 the data acquisition begins at time point T_start_ and is interrupted regularly to allow interlaced measuring of multiple samples.

When measuring multiple plants, each individual sample can be labeled with a temporal offset to its predecessor, and the plants are subsequently measured in a rotating scheme (Figure 2). The temporal distance (gap size *d*) between successive measurements of the same plant depends on the total number of plants measured, the length of the measurement window *w*, as well as the handling time T_h_ needed for transporting samples in and out of the measurement setup.

**Figure 3:**
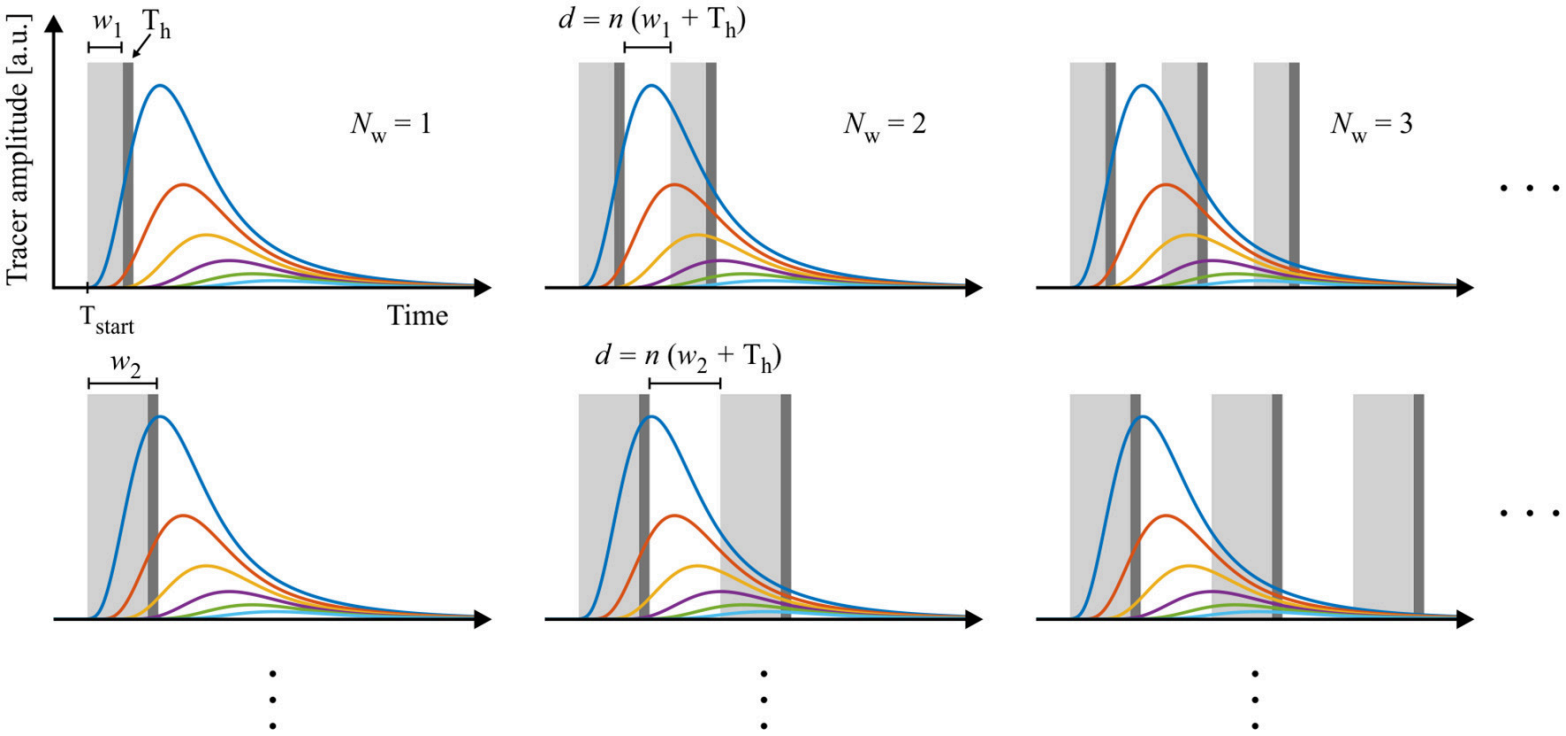
Schematic construction of measuring scenarios. Data are sampled with windows varying in starting time, frequency, length and distance between windows. The length of the gaps *d* between the windows are multiples of window width *w* plus handling time *T*_*h*_. In this way, all designs allow an interlaced measurement of multiple samples and an optimized use of measurement equipment.

An experimental design is then defined by the following experimental parameters: (1) starting time point T_start_, (2) window width *w*, (3) number of windows *N*_w_, (4) handling time T_h_, and (4) gap length between windows *d*. The gap *d* between two respective measurement windows is defined as a multiple of *w* + T_h_, ensuring that all further samples can be measured subsequently using the same experimental design. This procedure allows investigating the experimental design for one sample only which then represents all consecutive sample measurements. The construction of the experimental designs is depicted in Figure 3.

One of the main properties of each experimental design is the mean sample rate SR which represents the number of samples that can be measured per time unit. This sample rate is given by SR = (*N*_w_ · (*w* + T_h_))^−1^. The cumulated time of sample measurement can be calculated as *N*_w_ · *w* for each design.

## Case study

### Reference data

For this study a general reference data set was created with model M13 and artificial noise. A Gaussian distribution $\rho_{0}\left(x\right)=\exp\left(-{\left(x-x_{0}\right)}^{2}/2\sigma^{2}\right)$ served as a continuous initial tracer distribution function at time *t*_0_ = 0 with *x*_0_ as distance from the beginning of the experimental field of view at *x* = 0 and σ as width of the input curve. One of the major advantages of the spatial initial condition is the ability to deal with temporal gaps in the analyzed data and with tracer leaving the field of view, both cases in which a reconstruction of a temporal boundary condition is not possible. Also, implementing a spatial initial condition allows high convergence orders of the PDE solver at the boundaries of the field of view (Bühler et al., 2017). The parameters *x*_0_ and σ as well as the amplitude of the simulated tracer distribution within the field of view need to be fitted along with the model parameters. For fitting the model to experimental data, also the temporal position of the initial condition, *t*_0_, might need to be estimated, because a temporal shift of experimental data could affect the fit quality. Nevertheless, estimating *t*_0_ requires a continuous interpolation of the data to the shifted time grid. This interpolation can cause small numerical oscillations of the non-linear optimization near the minimum which can affect the comparison of different experimental designs. For this reason, *t*_0_ was set to zero and not fitted in this study.

**Figure 4:**
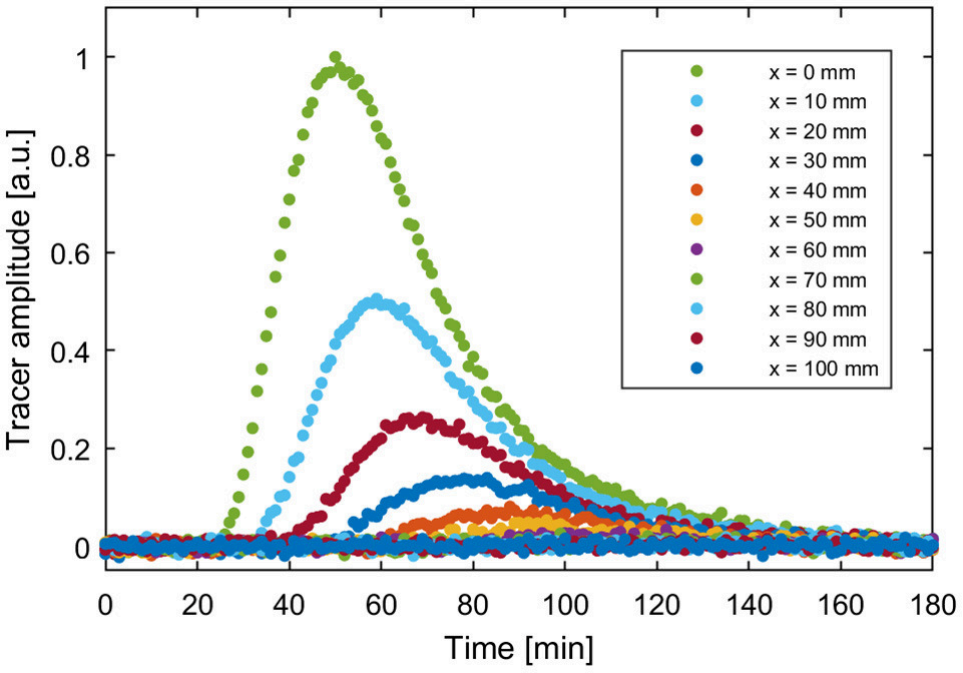
Example reference data set based on forward simulation of M13 with parameters ν = 2.0 mm min^−1^, *e*_12_ = 0.3 min^−1^, *e*_21_ = 0.1 min^−1^, *e*_23_ = 0.05 min^−1^, σ = 5 mm, *x*_0_ = 60 mm and added normally distributed noise with a standard deviation of 7e-3.

For the calculation of the reference data set, the parameter values of model M13 were set to *v* = 2.0 mm min^−1^, *e*_12_ = 0.3 min^−1^, *e*_21_ = 0.1 min^−1^, *e*_23_ = 0.05 min^−1^, and σ = 5 mm, *x*_0_ = 60 mm for the initial condition. The choice of model parameters was based on the fit parameters of model M13 to PET data of sugar beet in Bühler et al. (2014). The forward simulation was evaluated at 11 spatial positions from *x* = 0 to 100 mm in steps of 10 mm and with a temporal resolution of 1 min for a total time of 3 h. In order to simulate a certain model error, this data was repeatedly manipulated by adding normally distributed noise with a standard deviation of 7e–3. This value was chosen because the resulting standard errors of the estimated parameters are in the same range as the values for sugar beet in Bühler et al. (2014). The reference data set with one of the noise patterns used for all further calculations is shown in Figure 4.

### Specification of experimental designs

**Figure 5:**
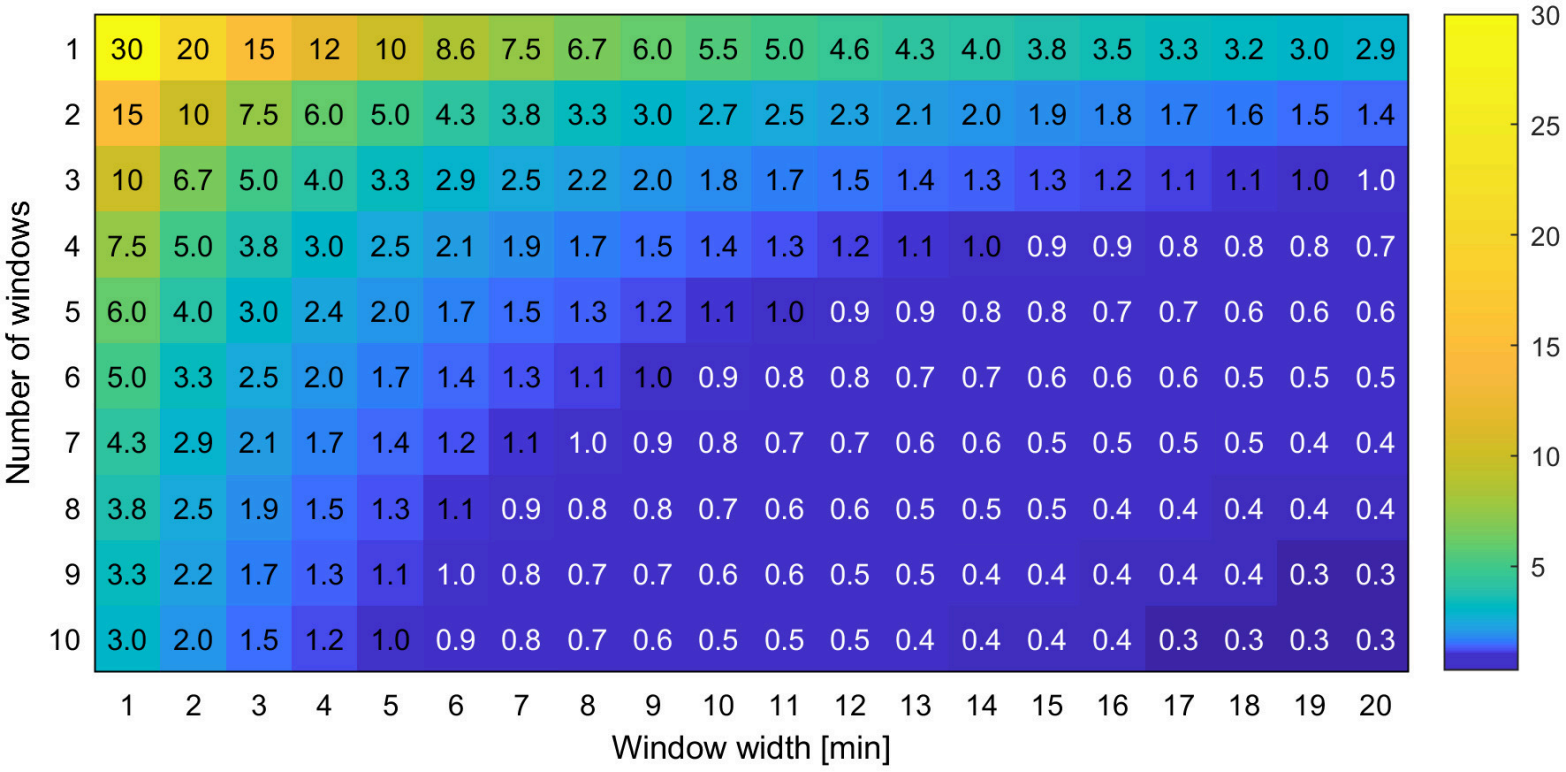
Exemplary view of the sample rate SR which is the number of samples measurable per hour for different experimental designs with varying number of windows and window widths.

The experimental designs described in section Experimental Design were applied on the reference data, resulting in reduced data sets, which are referred to as “designs” in the following. In order to make the number of possible designs feasible for calculations, the following side conditions were applied. Window widths *w* were considered in a range of values from 1 to 120 min in steps of 1 min. The starting time point T_start_ was varied in steps of 1 min from 20 to 40 min, i.e., starting data acquisition at the time of tracer arrival at the field of view and later. These conditions limited the maximal number of windows *N*_w_ to 26 which were iterated in steps of 1. The gap length *d* was then increased in multiples of the sum of window width *w* and handling time T_h_, until a maximum of around 120 min after starting the experiment was covered. For all calculations the handling time T_h_ was set to a constant value of 1 min. This procedure resulted in 12,547 different designs. Figure 5 shows the resulting sample rates SR for a subset of *N*_w_ = 1…10 and *w* = 1…20. For all calculations in the following, only sample rates ≥1.0 h^−1^ will be discussed. Areas with lower sample rates were not considered and were therefore labeled in white in Figure 5.

## Results and discussion

All 12,547 designs were fitted by model M13, taking as starting points the model parameters which were used for generating the reference data. The fitting results allowed the calculation of variances of the model parameter estimates for each design. Subsequently, the designs were filtered with regard to specific quality criteria, resulting in a set of possibly best designs for a given throughput. The filtered designs were further analyzed for sensitivity with regard to experimental variations in arrival time of tracer in the field of view. Finally, a general procedure for the generation of optimal experimental schedules was deduced.

### Error estimation of designs

**Figure 6:**
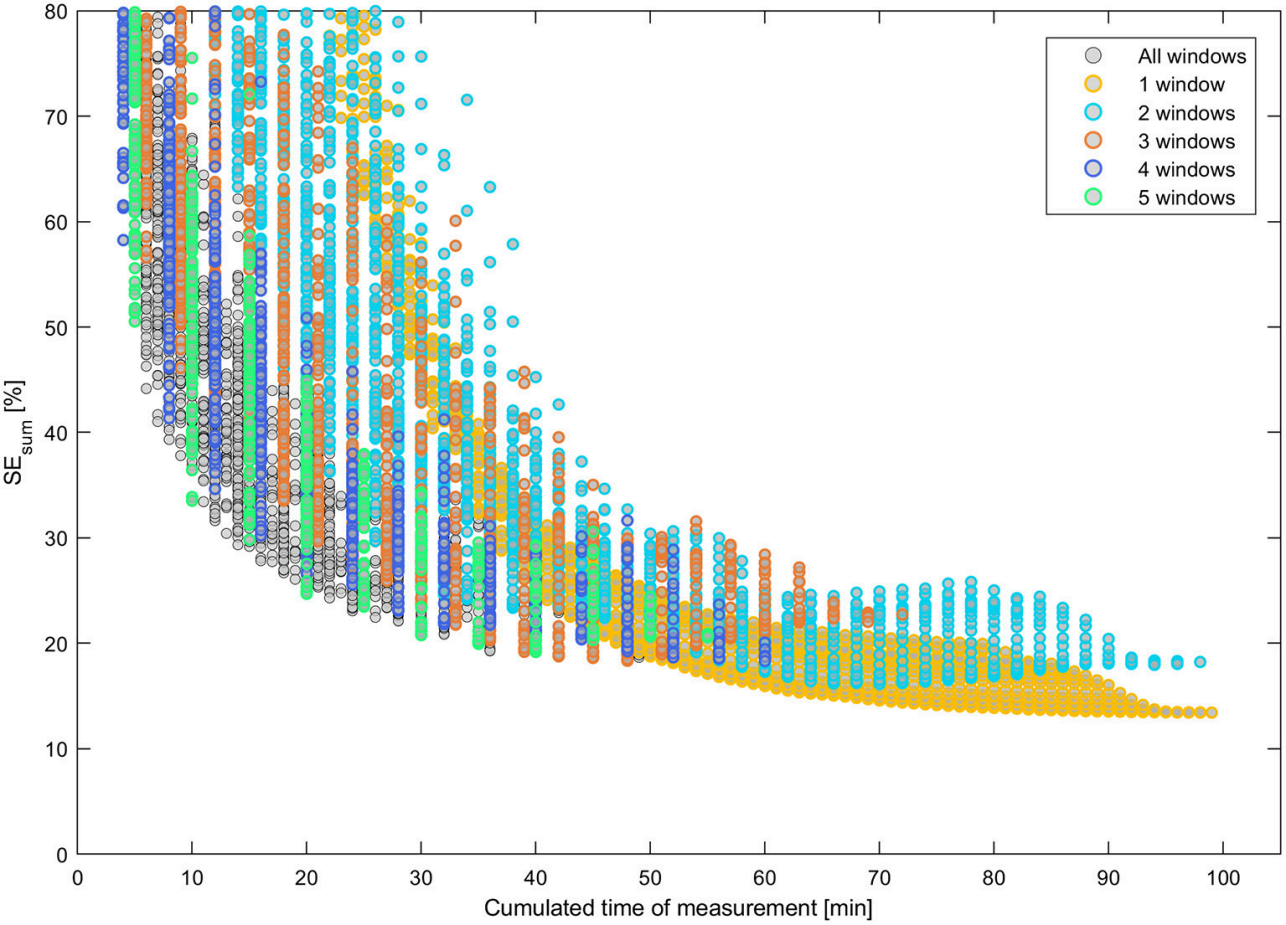
Uncertainty of model parameters SE_sum_ vs. cumulated time of measurement for all design with SE_sum_ ≤ 80%. Designs with a small number of windows are highlighted by colored rings, respectively.

The noise in the data caused the parameter estimates always to differ from the original underlying parameter set. The fitting was repeated five times for each design with different random noise and the fitting results were averaged. The definition of all designs and the respective fitting results can be found in Supplementary Table 2. The sum of the relative standard errors, SE_sum_, of the four most relevant model parameters (velocity *v* and exchange rates *e*_12_, *e*_21_, and *e*_23_) was used as a measure of the overall uncertainty of each design. This measure has the advantage to be independent of the different absolute parameter values. The standard errors are expected to roughly correlate with the cumulated time of measurement i.e., the longer the time of data acquisition, the lower the standard errors and, hence, the lower SE_sum_. Standard errors for the parameters *x*_0_ and σ of the initial function were not taken into account because the initial function is of purely auxiliary nature (Bühler et al., 2011). Figure 6 shows this relationship in detail for all designs up to a maximal SE_sum_ of 80%. Designs with a low number of windows *N*_w_ ≤ 5 are highlighted respectively by colored rings.

**Figure 7:**
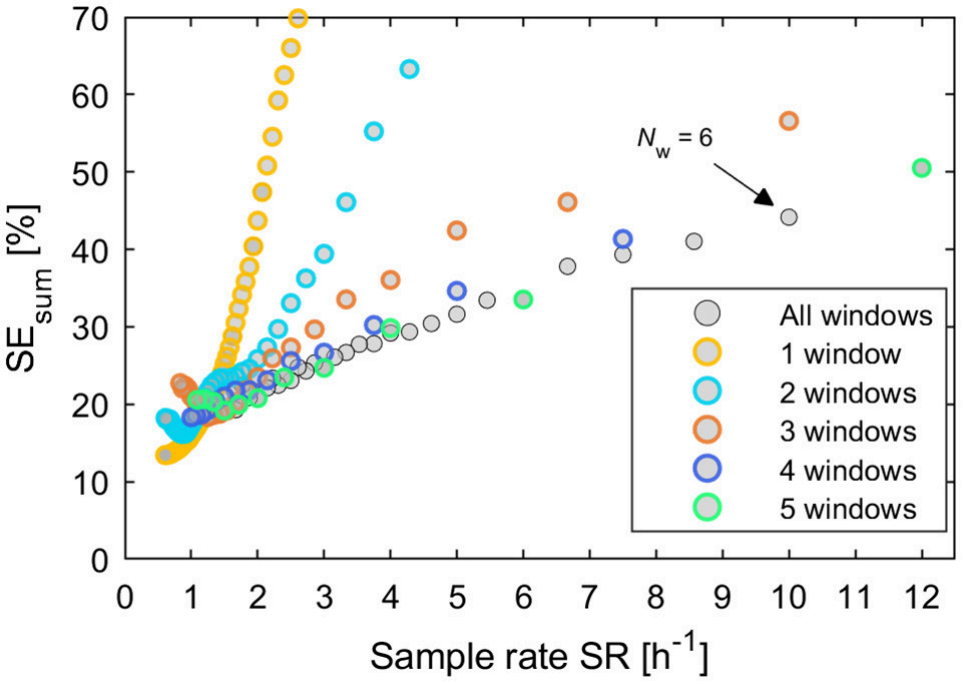
Plot of all potentially best designs from Figure 6 after filtering by the minimal sum of parameter uncertainties SE_sum_ for each unique value of cumulated measurement time. Additionally designs were included with a low number of windows between 1 and 5. These are highlighted by colored rings, respectively.

In Figure 6 some groups of continuous relations become apparent especially for designs with 1 and 2 windows. Nevertheless, of special interest are only designs that are located on the Pareto curve of a minimal SE_sum_ for each unique value of cumulated time of measurement. Additionally, all respective Pareto elements with a limited number of windows *N*_w_ ≤ 5 were considered, too. These minimal uncertainty designs were filtered out of the set of 12,547 designs and plotted vs. the respective sample rate SR in Figure 7. Here, designs with SE_sum_ higher than 70% are not regarded and, again, designs with a low number of windows *N*_w_ ≤ 5 are highlighted by colored rings. The maximal value of 12 samples per hour relates to a total measurement time of 5 min per sample.

For each number of windows, approximately linear relations of model uncertainties SE_sum_ to sample throughput are recognizable in Figure 7 for higher values of SR. Deviations from a clear linear trend could result from random patterns in data. The mean slope of each relation decreases with increasing number of windows up to *N*_w_ = 5. Designs with *N*_W_ > 5 do not significantly improve the relation between model uncertainties SE_sum_ and throughput SR any further. Designs with 2 windows cannot compete with any other designs. For low throughput with values < 1.2 samples h^−1^, designs with 1 window always prove to be an optimal choice. Above this value the selection of an optimal design becomes complex and designs with *N*_w_ ≥ 3 will be preferable.

### Selected designs

The following quality criteria for designs can be applied in order to filter optimal designs from the set of all possible designs. Any potentially best design

1. must provide a minimal value of SE_sum_ for its respective values of throughput SR and number of windows *N*_w_;
2. is preferred over another design if it has a lower number of windows *N*_w_ while throughput SR and uncertainty SE_sum_ remain similar;
3. allows a unique fitting to data for a preferably large range of starting parameter sets;
4. enables the identification of the underlying model;
5. shows a certain stability in SE_sum_ regarding shifts in T_start_ relative to the arrival time of tracer in the field of view.

Per definition, all pre-filtered designs shown in Figure 7 already fulfill quality criterion (1). By applying also quality criteria (2) to (4) to the pre-filtered designs, a set of 12 designs was selected covering sample rates from 1 to 12 samples h^−1^. The design definitions and corresponding values of SR and SE_sum_ are listed in Table 1. SE_sum_ correlates almost linearly with the noise amplitude (see Supplementary Figure 1) and, thus, the noise amplitude has no significant influence on the comparison of designs regarding parameter uncertainty. Validity of criterion (3) was confirmed by repeated successful fitting, starting with different parameter values far away from the minimum. Criterion (4) was ensured by comparing the results for fitting the designs with different selected model instances from the general model class, see Supplementary Material S5. SR ≥ 1 was assumed in order to ensure a significant improvement of throughput compared to the full reference data set. Designs with even higher sample rates than SR = 12 h^−1^ were not considered, because their corresponding high model uncertainties SE_sum_ of more than 50% severely limit their practical applicability.

**Table 1:** Resulting selected designs from application of quality criteria (1) to (4).

**Design**	***N*_w_**	***w***	**T_start_**	***d***	**SR**	**SE_sum_**
		**min**	**min**	**min**	**h**^−1^	**%**
1	1	60	24	–	1	15.95
2	3	13	25	14	1.54	19.19
3	5	6	25	7	2	20.78
4	5	5	25	6	2.4	23.47
5	5	4	23	10	3	24.70
6	4	4	23	10	3.75	30.23
7	5	3	23	12	4	29.84
8	4	3	23	12	5	34.64
9	5	2	25	12	6	33.53
10	4	2	25	12	7.5	41.34
11	6	1	25	10	10	44.16
12	5	1	25	12	12	50.52

*N_w_, number of windows; w, window width; T_start_, start time point of measurement; d, temporal distance between windows; SR, sample rate; SE_sum_, uncertainty measure. The sample handling time T_h_ is constant and set to 1 min for all designs*.

For the designs in Table 1, the relative standard errors of the single model parameters adding up to SE_sum_ increase uniformly with SE_sum_ (see Supplementary Table 2), but there are deviations from the proposed linear increase of SE_sum_ with increasing sample rate SR for designs 7 vs. 6 and 9 vs. 8. This results from applying criterion (2), by which designs for SR = 3.75 and SR = 5 with slightly lower SE_sum_, but much higher *N*_w_ were dismissed in favor of designs with *N*_w_ ≤ 5. Design 11 is the only exception from the approximate rule of *N*_w_ ≤ 5 for the selection in Table 1, included here because there was no other design matching the criteria in the range of 8 ≤ SR < 12 (see Figure 7 and Supplementary Table 2). The rule of *N*_w_ ≤ 5 was motivated by the fact that the specific handling time T_h_ = 1 min used in the calculations is a very rough estimate only. Handling time could be in the range of seconds for a, yet to build, efficient automated sample handling system and up to several minutes for manual sample handling. Also it is generally desirable to minimize disturbances of the samples by movements as well as to minimize manual handling of radioactively labeled samples by the experimentalist. Together, these arguments lead to the restriction of *N*_w_ applied in this study.

**Figure 8:**
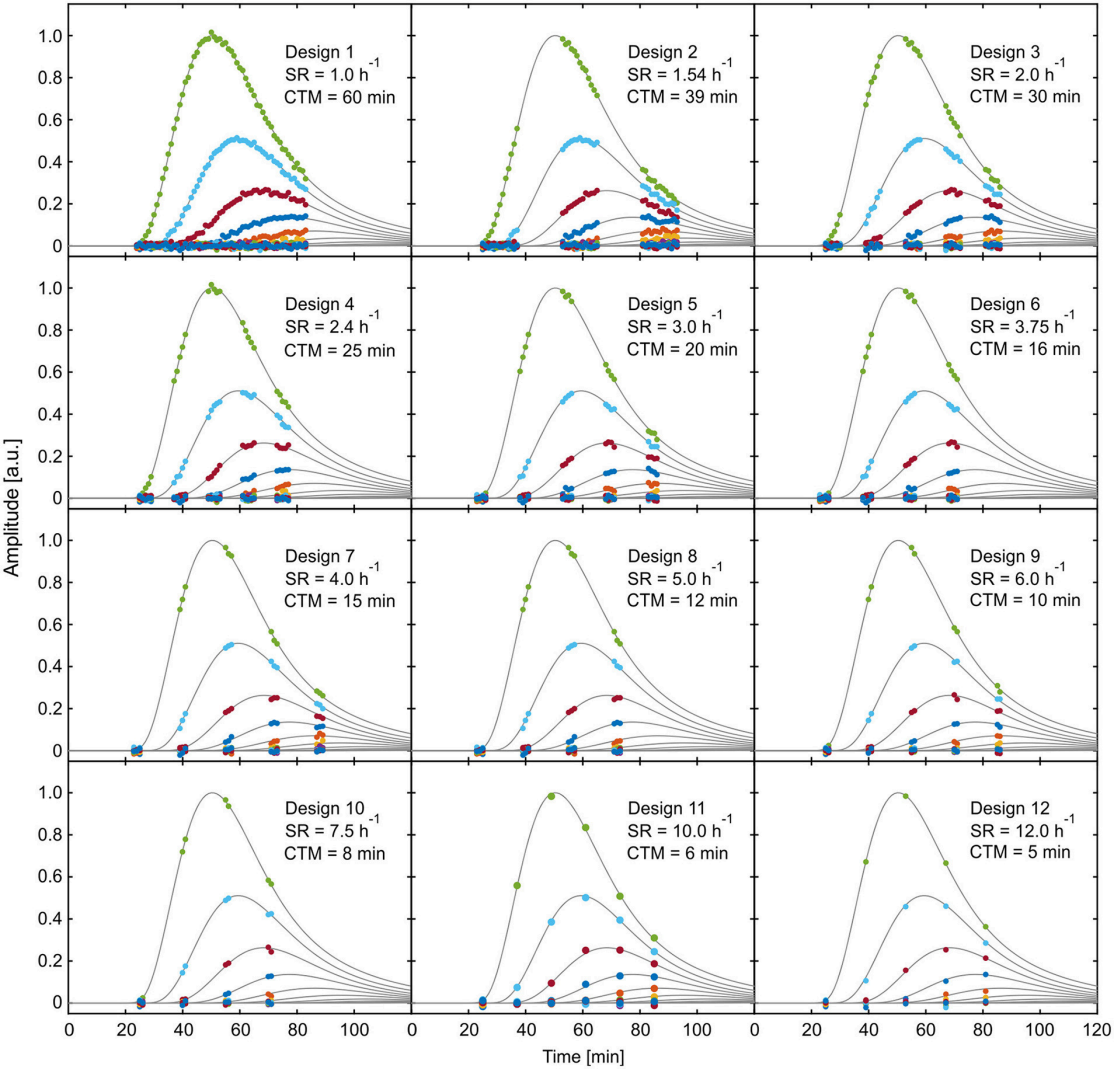
Visualization of exemplary reference data of selected experimental designs from Table 1. SR, sample rate; CTM, cumulated time of measurement.

The application of the selected designs to one of the reference data sets is visualized in Figure 8, illustrating the temporal distribution of the respective measurement windows. All these possibly best designs show certain common characteristics. For example, all designs start data acquisition between 23 and 25 min. This suggests that the rise of the leading curves in the reference data, corresponding to the arrival time of data in the field of view, contains important information which should always be covered. Also, almost all designs cover at least one of the peaks as well as the steepest areas of descent of one the dominant curves. The tail of the tracer profiles does not seem to contain essential information, since none of the possibly best designs includes data points after 93 min. Given that the time point T_start_, which marks the beginning of data acquisition, seems to be of crucial importance for the performance of all designs, criterion (5) will be investigated more thoroughly in the following.

### Optimal starting time points

**Figure 9:**
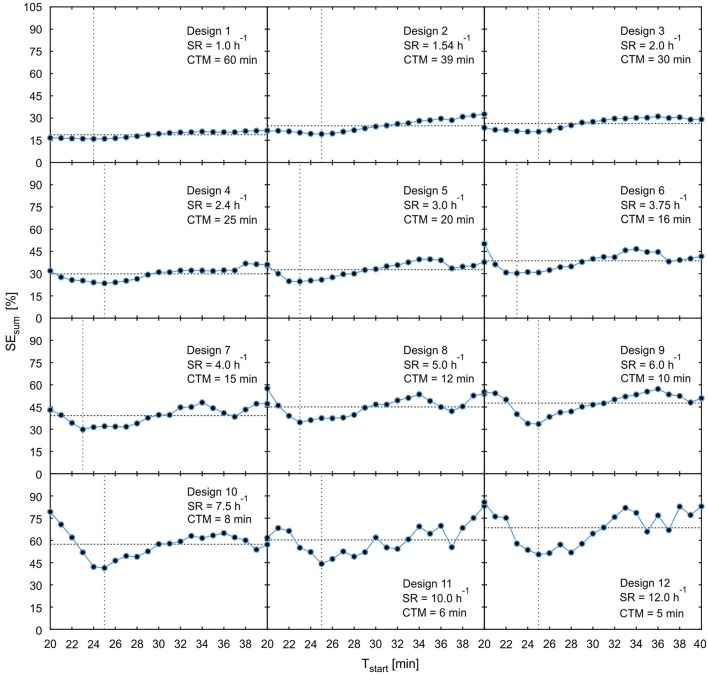
Plot of parameter uncertainty SE_sum_ for varying starting time points T_start_ starting from the 12 possibly best designs of Table 1. The horizontal dotted line shows mean value of SE_sum_, the vertical dotted line indicates time point of minimal SE_sum_. SR, sample rate; CTM, cumulated time of measurement.

For experimental setups with series of plants there will always be variations in the time point of tracer arrival in the field of view which are caused by structural biological differences between investigated samples. Therefore, experimental designs are preferable if they are less sensitive to variations in the starting time of data acquisition which relates to the time of tracer arrival in the field of view [criterion (5)]. In order to analyze these dependencies more closely, the model uncertainties SE_sum_ of all designs from Table 1 were plotted in Figure 9 with starting time points T_start_ varying from 20 to 40 min in steps of 1 min.

These plots reveal that designs with increasing throughput not only show higher minimal values of SE_sum_, but also consistently higher averages of SE_sum_ over the considered period of starting points and increasing sensitivity of SE_sum_ to variations of T_start_. For designs 9–12, the high sensitivity together with the lack of a plateau around the minimum is putting the practical applicability in doubt. In general, the time point of minimal SE_sum_ is not necessarily the best starting time point with regard to the sensitivity. For example, for designs 6–11, starting the data acquisition just 2–3 min earlier would cause significantly larger model uncertainties than starting some minutes after the minimum. This is caused by the fact that starting time points smaller than 20 min lead to data acquisition before tracer enters the field of view. Thus, these data windows contain less or no information at all about the tracer distribution, which causes an immediate increase of parameter uncertainties. In these cases, starting data acquisition some minutes later than at the minimum value of SE_sum_ would be a good strategy to avoid larger model uncertainties caused by biological variations in arrival time. In order to identify optimal starting points, pre-experiments with at least one non-interrupted measurement have to be performed in a specific experimental situation.

### General procedure for the identification of optimal designs

Pre-experiments will also be needed to determine the level of accuracy needed to enable identification of significant differences in an experimental study. A general procedure to arrive at optimal schedules will then consist of the following steps: (i) determination of a maximal uncertainty SE_sum_ of the parameter estimates depending on the required accuracy level in the experimental study; (ii) analysis of possible designs based on non-interrupted test-measurements, in the same way as presented in the preceding sections; (iii) selection of an optimal design with a maximal number of plants measureable per hour, depending on the maximal uncertainty SE_sum_. There will be no need to test all of the possible designs investigated in the present fundamental study. An adaption of the presented candidate designs to the actual shape of the tracer data is expected to be sufficient. As a general guideline, only such designs qualify as candidates for an optimal experimental design which cover the time point of tracer arrival in the field of view as well as the maxima and declining sections of the dominant first curves of the data set. Also, designs with 1 and 2 windows do not need to be considered for higher throughput scenarios. These constraints substantially reduce the number of candidates and enable a faster determination of optimal design for any given experimental setup.

For the case study, modeled instead of real data were used, which were known to represent real PET data of tracer transport in sugar beets very well (Bühler et al., 2014). The use of modeled data avoided obscuring the results by an (albeit tiny) model error, thus facilitating the systematic analysis. Since the presented case study represents only one exemplary experimental situation, two additional case studies with different transport properties were investigated as well. The results are available in Supplementary Materials S3, S4. In the case study based on tracer transport in maize root (Figure S3.1, parameter values from Bühler et al., 2014), the analysis was based on the simpler model M02 of the model class. This model consists of a transport—and a storage compartment only, without back-exchange of tracer. In the case study representing tracer transport in oak stems (Figure S4.1, parameter values based on De Schepper et al., 2013), model M13 was used, but with a lower spatial resolution, i.e., only 3 spatial positions instead of 11. As expected for different experimental setups, the level of SE_sum_ and the specific best designs for each sample rate differed for the different case studies. But qualitatively the results were very similar: calculation of SE_sum_ for all experimental designs (Figures S3.2, S4.2, in analogy to Figure 6) allowed the identification of possible best designs (Figures S3.3, S4.3, Tables S3.4, S4.4, in analogy to Figure 7 and Table 1), which could be further analyzed regarding variations of the starting time point T_start_ (Figures S3.5, S4.5, in analogy to Figure 9). Thus the general procedure works in all three cases, indicating that it is largely independent of the experimental setup as represented by the tracer transport properties and the spatial resolution of the measurements.

### Outlook

**Figure 10:**
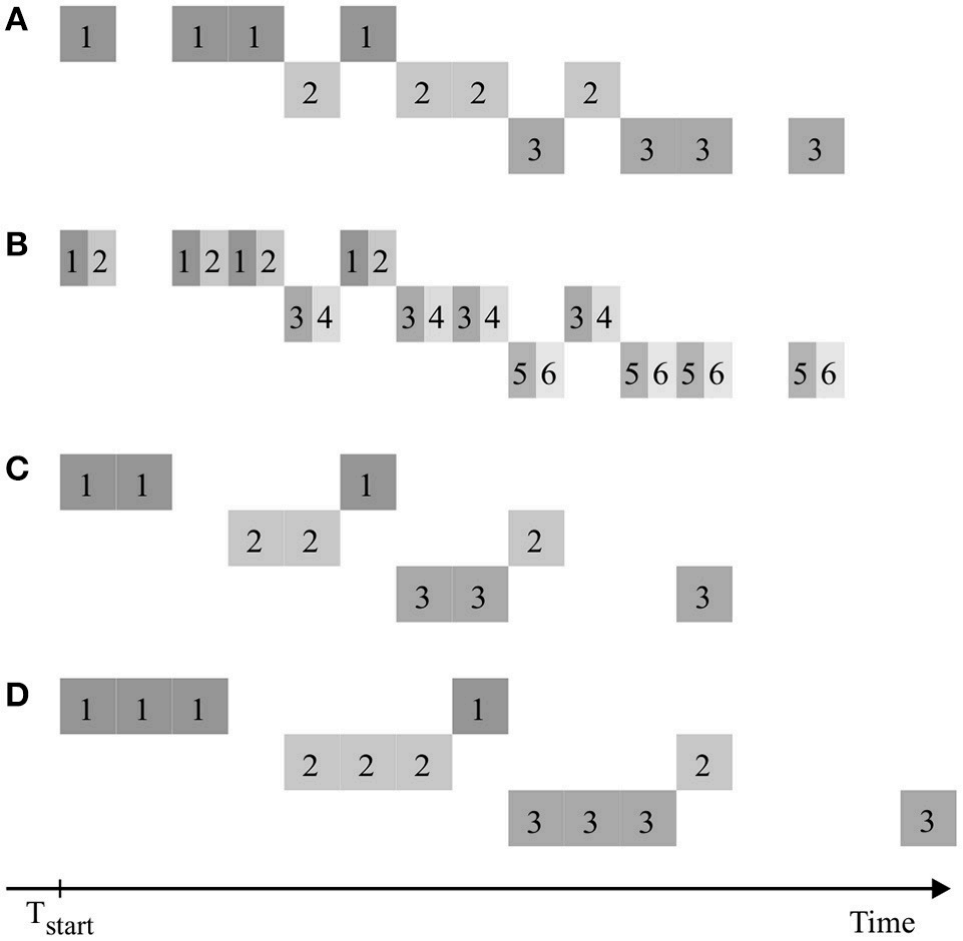
Examples of non-regular consecutive measuring patterns of multiple plant samples, denoted by numbers, in analogy to Figure 2. Scenarios **(A,B)** are symmetric, **(C,D)** are non-symmetric. The process of window splitting used to create **(B)** out of **(A)** can also be applied for **(C,D)**.

The quantitative results presented here depend on the specific properties of the chosen case study. Yet the workflow of design generation and evaluation can be applied analogously for other cases, e.g., with data from different plant species, or other spatial and temporal fields of view (possibly from other modalities of data acquisition than PET). The results could also be transferred to experimental setups using other isotopes than ^11^C, e.g., ^18^F with a half-life of 109.8 min (Partelová et al., 2014; Converse et al., 2015). Preliminary investigations, simulating the transport of ^18^F, showed a similar selection of possibly best designs, with a tendency of later optimal starting time points due to the longer half-life of this isotope. The construction of designs described in section Experimental Design could be extended by considering also non-regular patterns with different temporal distances between measurement windows. As for regular patterns, there should be no gaps between interlaced measurements of multiple samples in order to make full use of the measurement equipment. Figure 10 shows examples of symmetrical as well as non-symmetrical non-regular patterns meeting this requirement. From these basic patterns a set of 336 different designs was derived (Supplementary Table 2) and analyzed analogously to the regular pattern above. Though none of these designs showed any improvement over the corresponding regular designs with same throughput, this still might be the case for other non-regular patterns or experimental scenarios not considered so far.

## Conclusions

In this study the experimental design of interlaced labeling experiments of multiple samples for phenotyping internal transport mechanisms was investigated. A representative case study with a multitude of different designs was set up and evaluated with regard to accuracy of model-based analysis. For a range of different sample throughput scenarios from 1 to 12 samples h^−1^, there was always an optimal design. It is remarkable that in high throughput scenarios the model parameters could still be reconstructed with quite a small number of data points.

The presented procedure of design construction and evaluation serves as a framework for developing optimal experimental schedules by increasing sample throughput and at the same time keeping a required statistical reliability or, the other way around, minimizing the statistical uncertainty for a required throughput. For practical applications with a specific experimental setup, the maximal number of plants measureable per hour will not only depend on the required accuracy, but also on sample handling time. Before starting a series of interlaced plant experiments it will be necessary to perform thorough preliminary investigations on the plant species or genotypes of interest and test the presented procedure of design evaluation on complete data sets in order to find respective best case designs.

## Author contributions

JB, EvL, and GH designed the study; JB and GH performed the research; all authors contributed to writing the manuscript and approved the submitted version.

### Conflict of interest statement

The authors declare that the research was conducted in the absence of any commercial or financial relationships that could be construed as a potential conflict of interest.

## Supplementary material

Supplementary Figure 1 Dependence of parameter uncertainty SE_sum_ on noise. Noise level is defined as the standard deviation of added normally distributed noise.

Supplementary Table 2 Data table including all design definitions and fit results.

Supplementary Material S3 Results of additional case study based on maize root transport properties.

Supplementary Material S4 Results of additional case study based on oak stem transport properties.

Supplementary Material S5 Model identifiability.
