# Supplementary material for: Model-Based Design of Long-Distance Tracer Transport Experiments in Plants
Source: Front Plant Sci. 2018 Jun 7;9:773. doi: 10.3389/fpls.2018.00773 (PMC6001040; doi:10.3389/fpls.2018.00773)
Supplement: Supplementary Figure 1 — Dependence of parameter uncertainty SEsum on noise. Noise level is defined as the standard deviation of added normally distributed noise. [file Image_1.pdf]

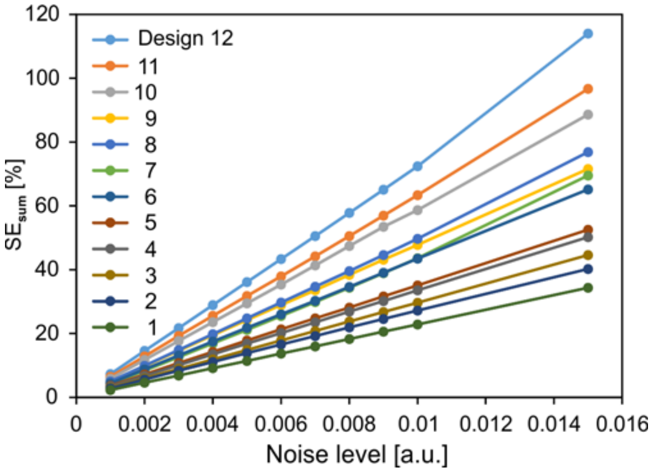

Figure S1. Dependence of parameter uncertainty SE_{sum} on noise. Noise level is defined as the standard deviation of added normally distributed noise.
