# Supplementary material for: Model-Based Design of Long-Distance Tracer Transport Experiments in Plants
Source: Front Plant Sci. 2018 Jun 7;9:773. doi: 10.3389/fpls.2018.00773 (PMC6001040; doi:10.3389/fpls.2018.00773)
Supplement: Supplementary Material S3 — Results of additional case study based on maize root transport properties. [file Data_Sheet_3.ZIP › Supplementary Figure S3.1.pdf]

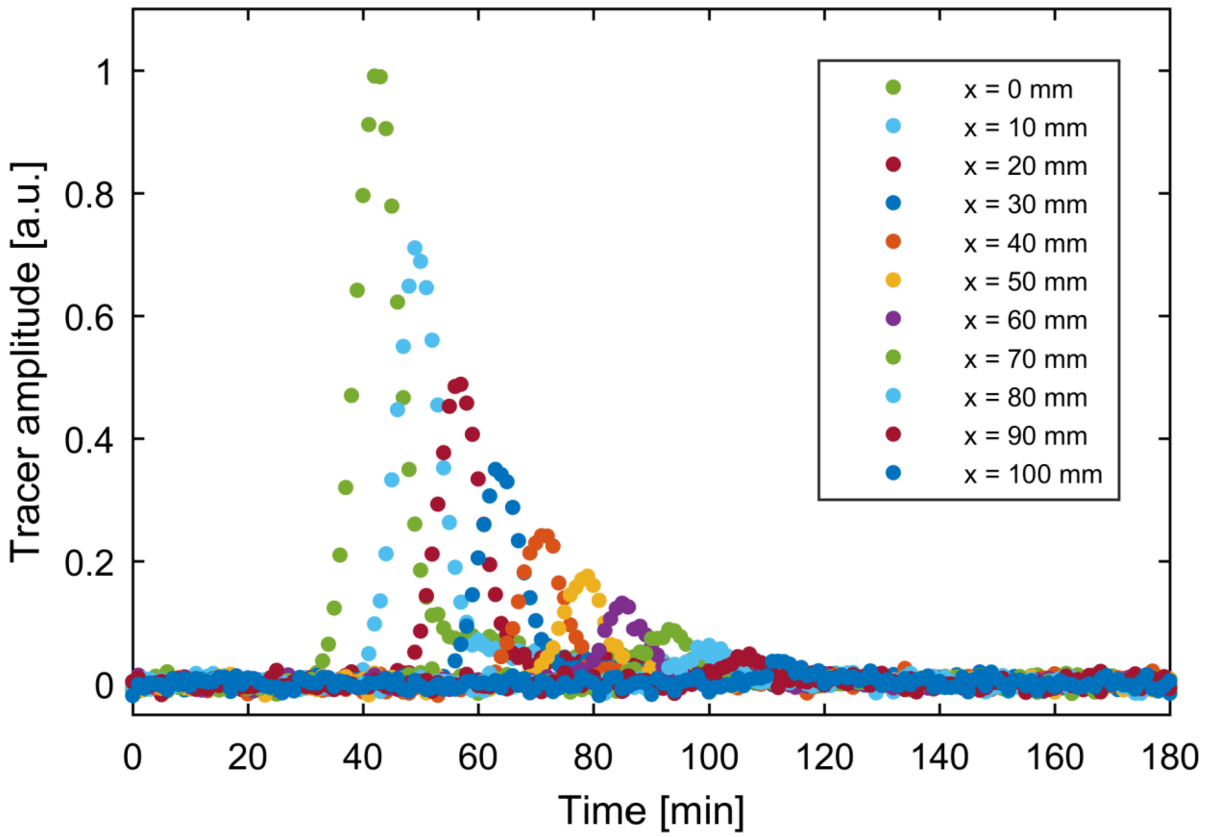

Supplementary Figure S3.1.
Supplementary reference data set based on the forward simulation of model M02 of Bühler et al. (2014), which is a simple two-compartment model with transport and storage, but no reloading. Model parameters were chosen based on the fit parameters of model M02 to PET data of maize roots (Bühler et al., 2014) as $v = 1.4$ mm min^{-1}, $e_{12} = 0.015$ min^{-1}, $\sigma = 5$ mm, and $x_0 = 60$ mm. Normally distributed noise was added with a standard deviation of 7e-3.
