# Supplementary material for: Model-Based Design of Long-Distance Tracer Transport Experiments in Plants
Source: Front Plant Sci. 2018 Jun 7;9:773. doi: 10.3389/fpls.2018.00773 (PMC6001040; doi:10.3389/fpls.2018.00773)
Supplement: Supplementary Material S3 — Results of additional case study based on maize root transport properties. [file Data_Sheet_3.ZIP › Supplementary Figure S3.2.pdf]

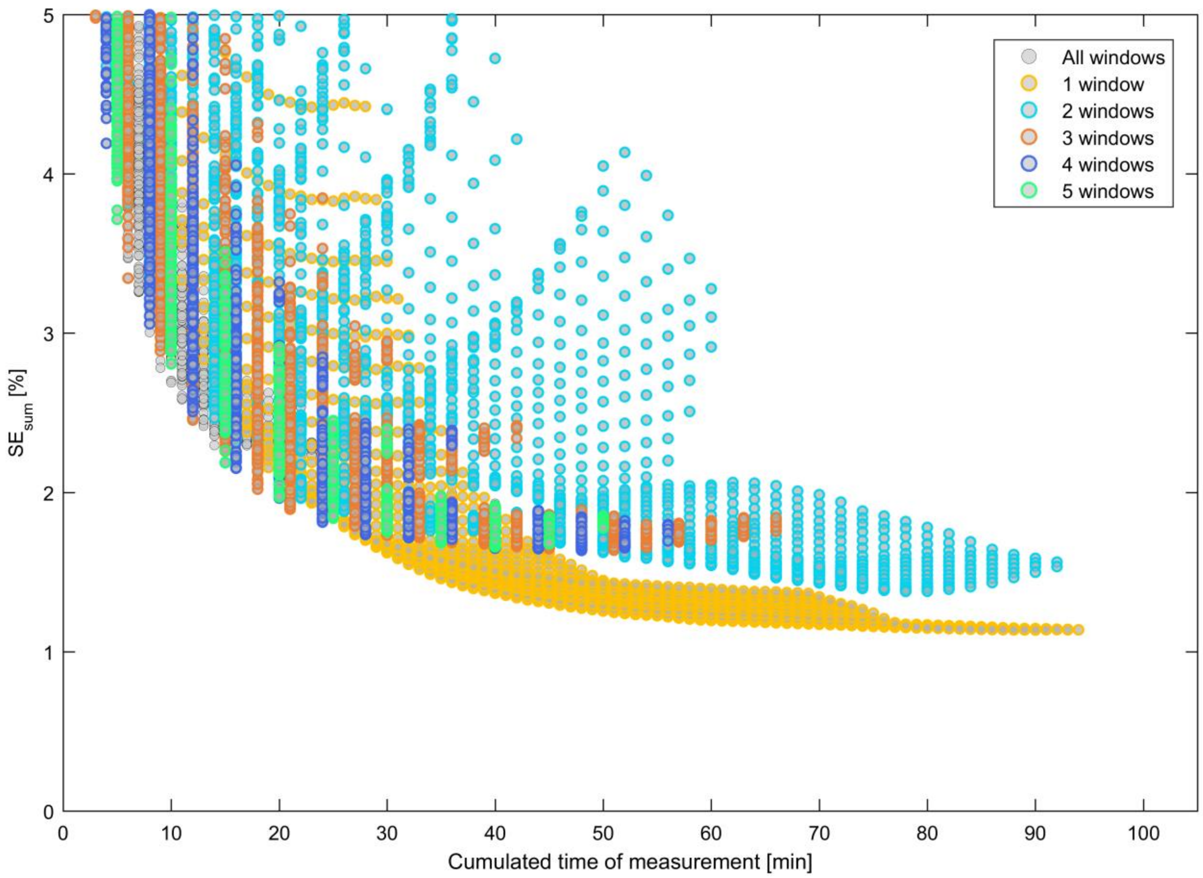

Supplementary Figure S3.2.

Uncertainty of model parameters SE_{sum} vs. cumulated time of measurement for all design with $SE_{sum} \leq 5\%$, based on the reference data from Supplementary Figure S3.1. Designs with a small number of windows are highlighted by colored rings, respectively.
