# Supplementary material for: Model-Based Design of Long-Distance Tracer Transport Experiments in Plants
Source: Front Plant Sci. 2018 Jun 7;9:773. doi: 10.3389/fpls.2018.00773 (PMC6001040; doi:10.3389/fpls.2018.00773)
Supplement: Supplementary Material S3 — Results of additional case study based on maize root transport properties. [file Data_Sheet_3.ZIP › Supplementary Figure S3.5.pdf]

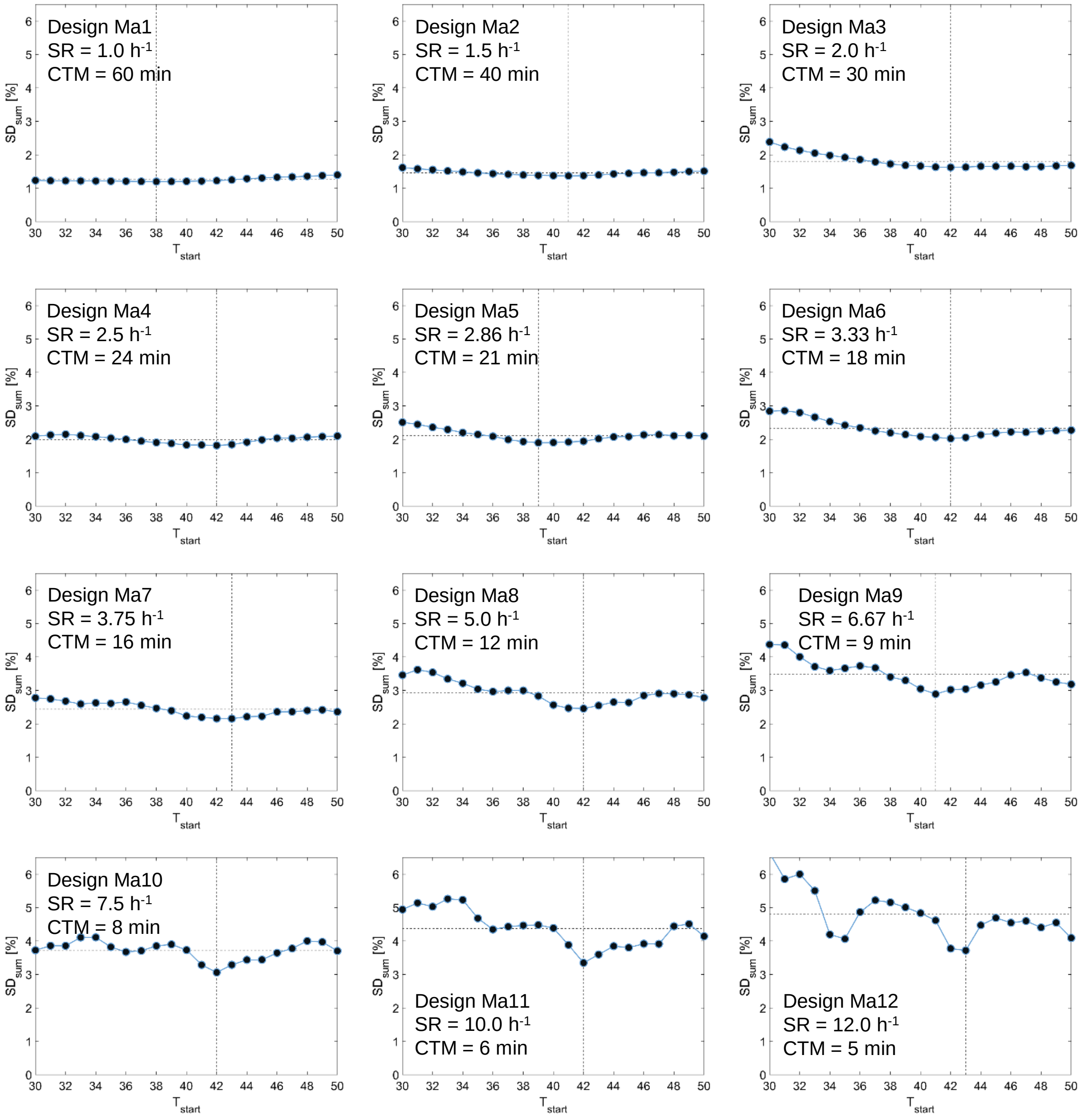

Supplementary Figure S3.5.

Plot of parameter uncertainty SE_{sum} for varying starting time points T_{start} starting from the 12 possibly best designs of Supplementary Table S3.4. The horizontal dotted line shows mean value of SE_{sum}, the vertical dotted line indicates time point of minimal SE_{sum}. SR = sample rate, CTM = cumulated time of measurement.
