# Supplementary material for: Model-Based Design of Long-Distance Tracer Transport Experiments in Plants
Source: Front Plant Sci. 2018 Jun 7;9:773. doi: 10.3389/fpls.2018.00773 (PMC6001040; doi:10.3389/fpls.2018.00773)
Supplement: Supplementary Material S3 — Results of additional case study based on maize root transport properties. [file Data_Sheet_3.ZIP › Supplementary Table S3.4.pdf]

Table S3.4.
Resulting selected designs from application of quality criteria (1) to (4) to the designs of Supplementary Figure S3.2. N_w = number of windows, w = window width, T_{start} = start time point of measurement, d = temporal distance between windows, SR = sample rate and SE_{sum} = uncertainty measure. The sample handling time T_h is constant and set to 1 minute for all designs.

Design	N_w	w	T_{start}	d	SR	SE_{sum}
		min	min	min	h^{-1}	%
Ma1	1	60	38	-	1	1.20
Ma2	1	40	41	-	1.5	1.37
Ma3	1	30	42	-	2	1.62
Ma4	4	6	42	7	2.5	1.81
Ma5	3	7	39	8	2.86	1.90
Ma6	3	6	42	7	3.33	2.02
Ma7	4	4	43	5	3.75	2.15
Ma8	3	4	42	10	5	2.45
Ma9	3	3	41	12	6.67	2.89
Ma10	4	2	42	12	7.5	3.06
Ma11	3	2	42	12	10	3.34
Ma12	5	1	43	6	12	3.71
