# Supplementary material for: Model-Based Design of Long-Distance Tracer Transport Experiments in Plants
Source: Front Plant Sci. 2018 Jun 7;9:773. doi: 10.3389/fpls.2018.00773 (PMC6001040; doi:10.3389/fpls.2018.00773)
Supplement: Supplementary Material S4 — Results of additional case study based on oak stem transport properties. [file Data_Sheet_4.ZIP › Supplementary Figure S4.1.pdf]

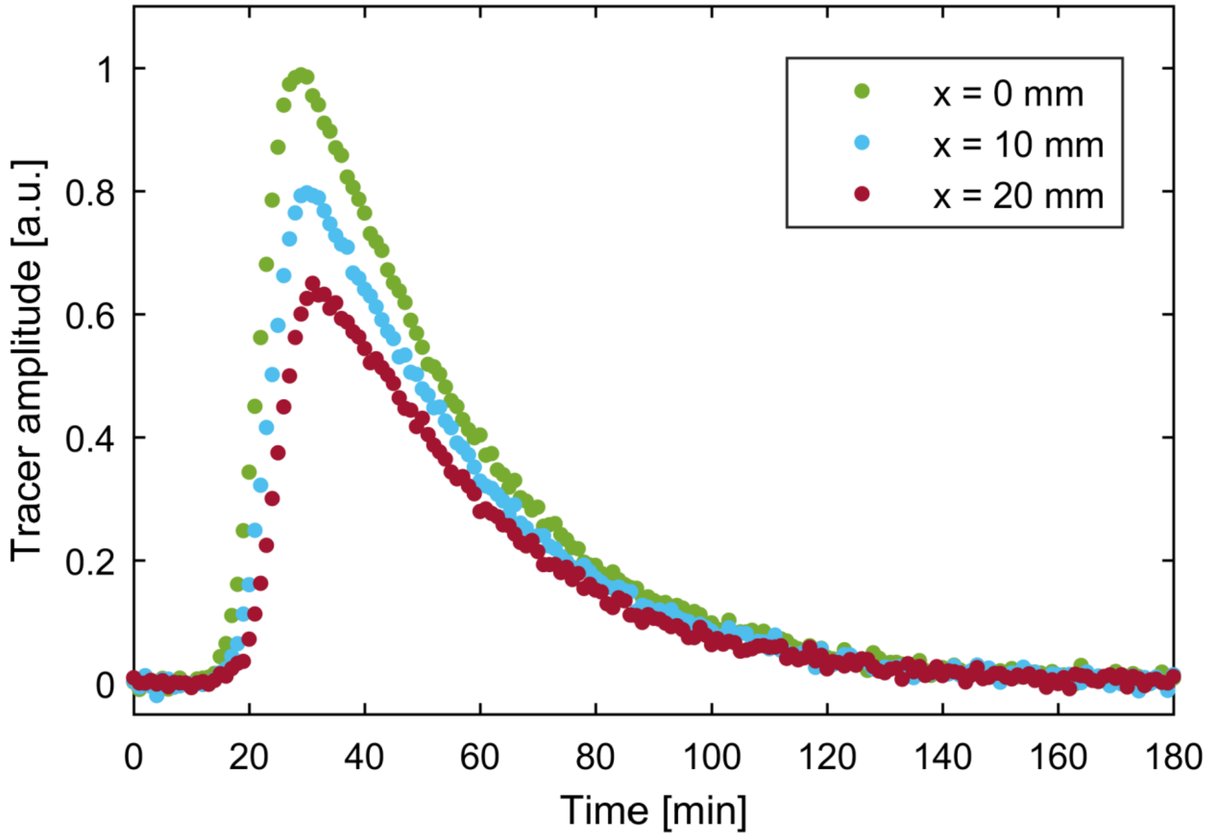

Supplementary Figure S4.1.
Supplementary reference data set based on the forward simulation of model M13 of Bühler et al. (2014) with model parameters v = 7.36 mm min^{-1}, e_{12} = 0.155 min^{-1}, e_{21} = 0.043 min^{-1}, e_{23} = 0.082 min^{-1}, σ = 30 mm, and x_0 = 200 mm. These values are based on the fit parameters of model M13 to PET data of oak stems (De Schepper et al., 2013). Normally distributed noise was added with a standard deviation of 7e-3.
