# Supplementary material for: Model-Based Design of Long-Distance Tracer Transport Experiments in Plants
Source: Front Plant Sci. 2018 Jun 7;9:773. doi: 10.3389/fpls.2018.00773 (PMC6001040; doi:10.3389/fpls.2018.00773)
Supplement: Supplementary Material S4 — Results of additional case study based on oak stem transport properties. [file Data_Sheet_4.ZIP › Supplementary Figure S4.3.pdf]

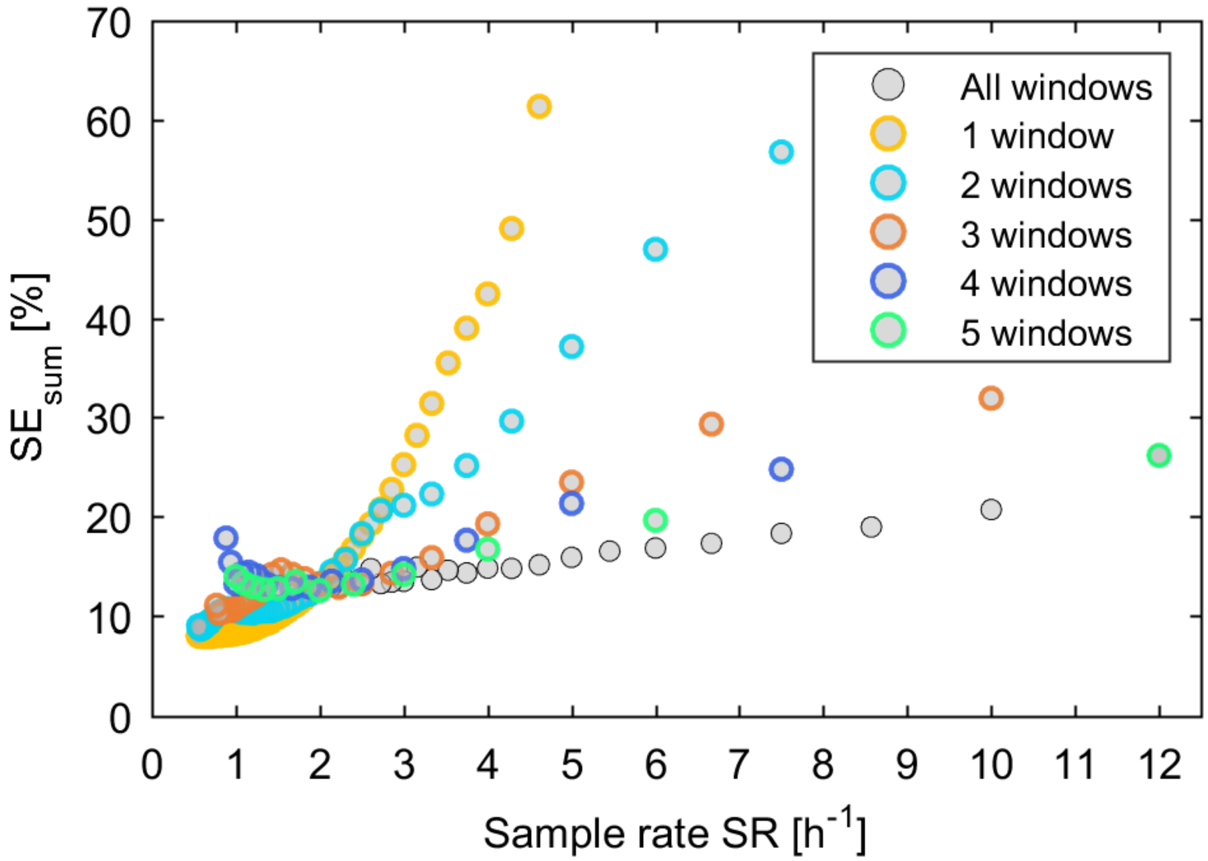

Supplementary Figure S4.3.
Plot of all potentially best designs from Supplementary Figure S4.2 after filtering by the minimal sum of parameter uncertainties SE_{sum} for each unique value of cumulated measurement time. Additionally designs were included with a low number of windows between 1 and 5. These are highlighted by colored rings, respectively.
