# Supplementary material for: Model-Based Design of Long-Distance Tracer Transport Experiments in Plants
Source: Front Plant Sci. 2018 Jun 7;9:773. doi: 10.3389/fpls.2018.00773 (PMC6001040; doi:10.3389/fpls.2018.00773)
Supplement: Supplementary Material S4 — Results of additional case study based on oak stem transport properties. [file Data_Sheet_4.ZIP › Supplementary Table S4.4.pdf]

Table S4.4.

Resulting selected designs from application of quality criteria (1) to (4) to the designs of Supplementary Figure S4.2. N_w = number of windows, w = window width, T_{start} = start time point of measurement, d = temporal distance between windows, SR = sample rate and SE_{sum} = uncertainty measure. The sample handling time T_h is constant and set to 1 minute for all designs.

Design	N_w	w	T_{start}	d	SR	SE_{sum}
		**min**	**min**	**min**	**h^{-1}**	**%**
O1	1	60	18	-	1	8.34
O2	1	40	16	-	1.5	9.84
O3	1	32	20	-	1.875	12.16
O4	3	8	20	9	2.5	13.22
O5	5	4	16	5	3	14.17
O6	5	3	21	4	4	16.73
O7	5	2	20	6	6	19.63
O8	6	1	20	6	10	20.71
O9	5	1	16	8	12	26.18
