# Supplementary material for: Model-Based Design of Long-Distance Tracer Transport Experiments in Plants
Source: Front Plant Sci. 2018 Jun 7;9:773. doi: 10.3389/fpls.2018.00773 (PMC6001040; doi:10.3389/fpls.2018.00773)
Supplement: Supplementary Material S5 — Model identifiability. [file Data_Sheet_5.docx]

# Supplementary Material S5. Model identifiability

In the context of this study model identification means that the best reconstruction of a given set of data can be achieved only with the original model that was used to create the data set. On the one hand, more complex models than the original model might be able to fit the data similarly well but they will probably be over-parameterized, resulting in significantly larger standard errors of the estimated model parameters. For this reason such cases are not considered here. On the other hand, models with less parameters than the original model should not allow an equally good data representation. Reducing the reference data sets by applying the experimental designs will always cause a certain loss of information. In the worst case, the reduced data sets might contain so little information that a fit with a simpler model produces a similar data representation as the original model.

In order to ensure that all possibly best designs from Table 1 allow an identification of the original model M13 these designs were fitted using two other models from the model class, model M02 with two parameters and M05 with three parameters (Figure S5.1). These models were chosen because they are the only models of the model family which can be derived from M13 by eliminating one or two parameters, respectively (see Figures 2 and 3 in Bühler et al. (2014)).


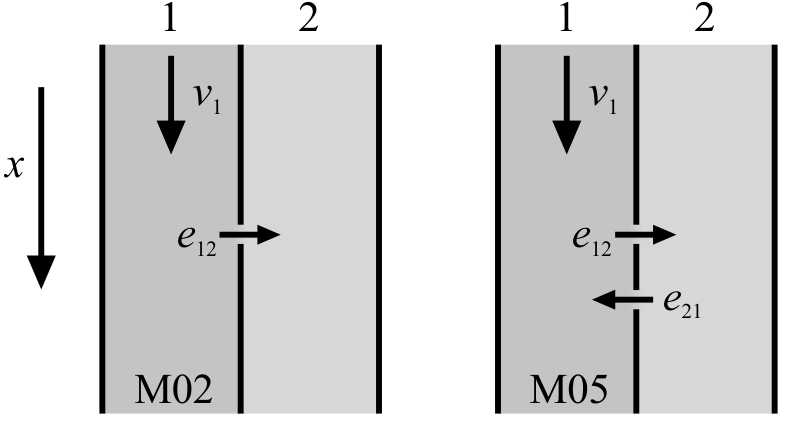


**Figure S5.1.** Sketch of models M02 and M05 from the model family in Bühler et al. (2014).

Figure S5.2 shows the values of the variances *s*^2^, i.e. the squared differences of the data and the simulated data divided by the number of data points, for all designs from Table 1 fitted with models M02, M05 and M13. For all designs, *χ*² decreases with higher model complexity, and model M13 constantly shows by far the best values for *χ*². Even for the high-throughput designs 10-12 there is no indication of over-parameterization of M13.


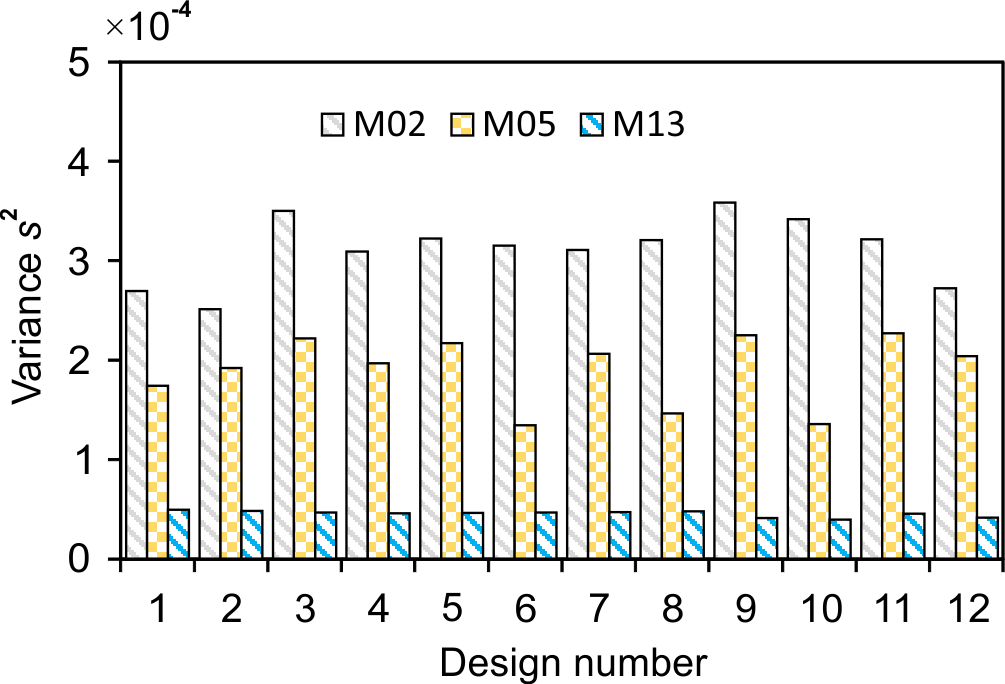


**Figure S5.2.** Variances *s*^2^ for models M02, M05 and M13 fitted to all 12 designs from Table 1.

Figure S5.3 shows the mean values for the relative standard errors of the model parameters, SE_mean_, for all designs from Table 1 fitted with models M02, M05 and M13. SE_mean_ is defined as SE_mean_ = SE_sum_ / (number of model parameters), allowing a comparison of the standard errors between models with different number of parameters. For all models the values for SE_mean_ are in a similar order of magnitude for each design respectively. This also demonstrates that model M13 is not over-parameterized for any of the selected possibly best designs of Table 1.


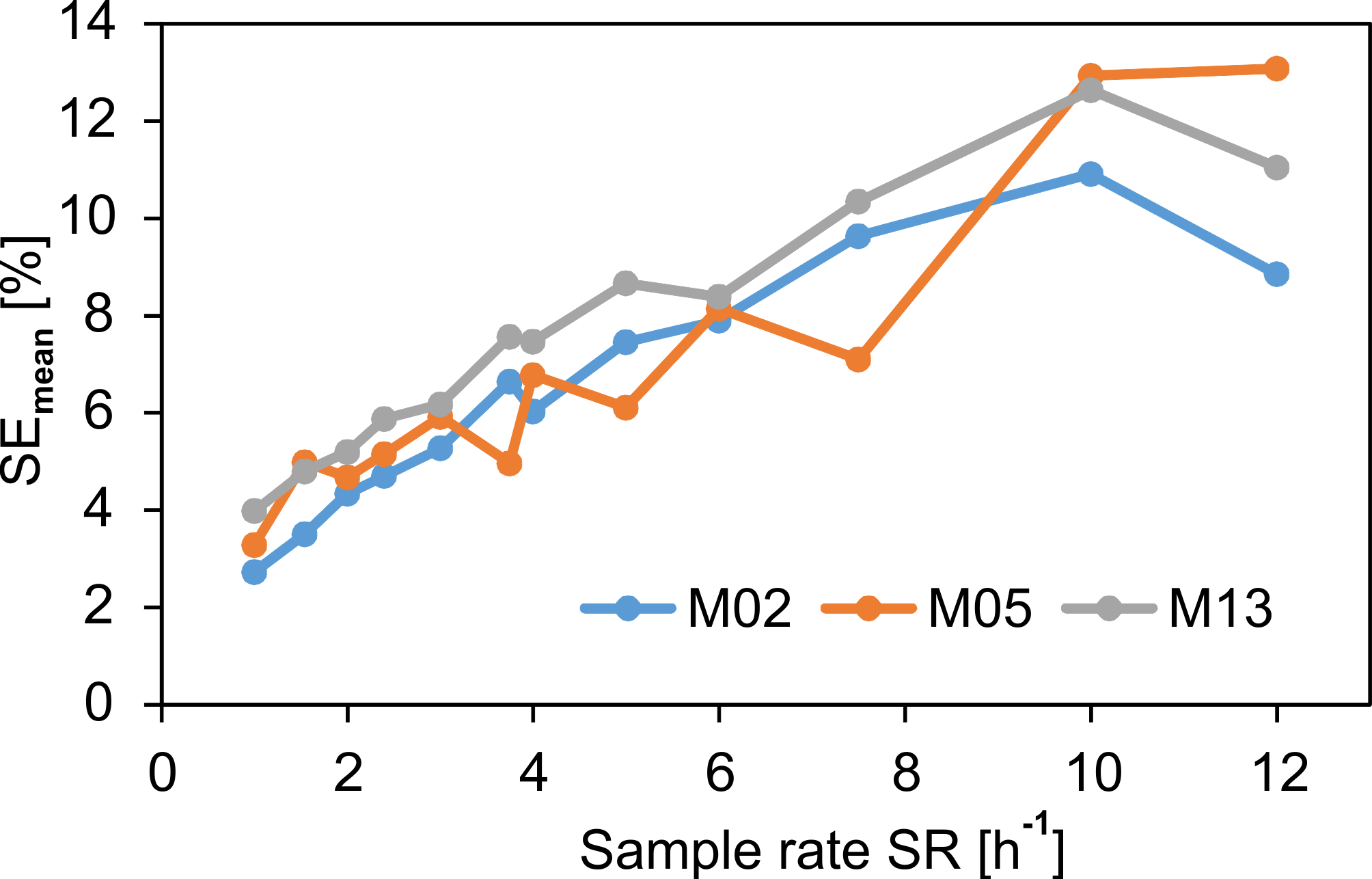


**Figure S5.3.** Mean parameter uncertainty SE_mean_ depending on sample rate SR for models M02, M05 and M13 fitted to all 12 designs from Table 1.
